# Beyond Clots: The Role of Cardiac Anesthesiologists in a Case of ECMO for Massive Pulmonary Embolism

**DOI:** 10.1155/crcc/5573593

**Published:** 2025-06-22

**Authors:** Vimal Varma, Isqandar Adnan, Engku Naim, Rusnaini Mustapha

**Affiliations:** Department of Cardiothoracic Anaesthesia & Intensive Care, Faculty of Medicine, Universiti Teknologi MARA (UiTM), Sungai Buloh, Selangor, Malaysia

**Keywords:** cardiac anesthesiologist, catheter-directed mechanical thrombectomy (CDMT), extracorporeal membrane oxygenation (ECMO), pulmonary embolism

## Abstract

We present a case of a 35-year-old male who presented with sudden-onset severe chest pain and breathlessness. CTPA revealed massive bilateral pulmonary embolism. The patient underwent catheter-directed mechanical thrombectomy (CDMT) under general anesthesia, complicated by cardiogenic shock necessitating cardiopulmonary resuscitation (CPR). Post ROSC, venoarterial ECMO was initiated, and catheter-directed thrombolysis was performed. CDMT was reattempted but not fully successful, and the patient developed right heart failure with pulmonary hypertension. ECMO continued for 5 days and gradually weaned off. He was extubated successfully on Day 7 and discharged home later.

## 1. Introduction

Extracorporeal membrane oxygenation (ECMO) is a life-saving intervention for patients with severe cardiac and/or pulmonary dysfunction unresponsive to conventional medical therapies. Patients on ECMO require vigilant care in ventilation, sedation, hemodynamics, anticoagulation, nutritional support, sepsis control, and multiorgan support. Anesthesiologists involved in cardiothoracic critical care play a multifaceted role, integrating multidisciplinary teams to manage patients on ECMO and ensure optimal outcomes.

## 2. Clinical Presentation

A 35-year-old male, school teacher, presented with sudden onset of severe chest pain at rest and breathlessness. He started experiencing mild reduced effort tolerance for the past 3 days. Otherwise, he denied any history of failure symptoms. He is a chronic smoker with a BMI of 32. He has a history of gouty arthritis for which he has been consuming traditional medication/supplements in the form of juice. His father had a history of ischemic heart disease. There is no history of cancer, blood disorders, recent major surgeries, long travel, or immobilization.

Upon presentation, he was conscious and alert; physical examinations were unremarkable with Spo2 97% under room air and tachycardia of 110 beats per minute.

ECG showed sinus tachycardia with Wellen's pattern. The echocardiogram showed a left ventricular ejection fraction of 45% with the right ventricle and right atrium dilated with estimated pulmonary artery systolic pressure (PASP) of 54 mmHg. Blood investigations showed raised troponin I and pro-BNP. ACS treatment started and proceeded with a coronary angiogram which showed normal findings. ABG showed Type 1 respiratory failure with PaO2 of 66 mmHg. He was put on nasal prong oxygen at 3 L/min.

**Figure 1:**
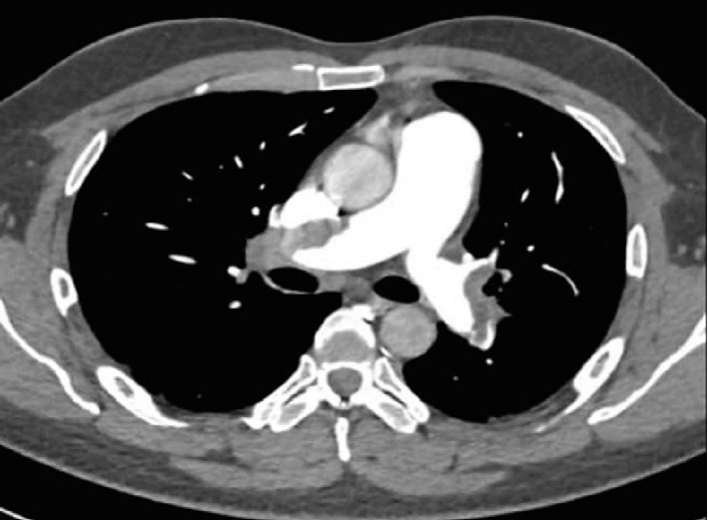
CT angiography of pulmonary artery (CTPA) revealed large filling defects in the right and left pulmonary arteries extending into segmental and subsegmental branches.

Given persistent sinus tachycardia and hypoxemia, computed tomography pulmonary angiography (CTPA) was done revealing large filling defects seen in the right and left main pulmonary artery (PA). The filling defects extend into the interlobar and the upper, middle (left), and lower lobes' segmental and subsegmental branches (Figure 1).

The cardiology team planned for catheter-directed mechanical thrombectomy (CDMT) using a Penumbra system under general anesthesia for bilateral massive PE.

The patient was electively intubated for the procedure. The right internal jugular venous and right radial arterial access were obtained before induction. The patient was hemodynamically stable after induction.

Percutaneous mechanical aspiration thrombectomy was performed via the right femoral vein approach using the Penumbra system. A pulmonary angiogram revealed large filling defects in the right and left pulmonary arteries. Clots were successfully aspirated from the right PA. However, the cardiologist had difficulty wiring and aspirating the left PA thrombus, during which the patient developed malignant tachyarrhythmia and PEA needing CPR. The patient developed ROSC after 2 cycles of CPR, defibrillation, and 1 mg of adrenaline. Post ROSC, he required four inotropes (noradrenaline, adrenaline, vasopressin, and milrinone) and high ventilator settings. Bedside ECHO (TTE) revealed poor RV and LV contractility with dilated RV.

The cardiothoracic anesthesiologist suggested ECMO after discussing with the cardiologist, and a referral was made to the cardiothoracic surgical team.

**Figure 2:**
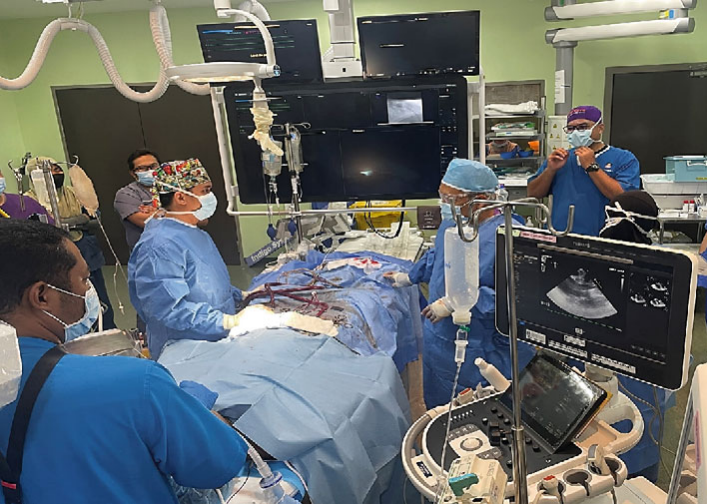
Cardiac anesthesiologist performing transesophageal echocardiography (TOE) to confirm the placement of ECMO cannulas.

Venoarterial (V-A) ECMO was agreed upon by all teams. Right-sided femoral artery and venous cannulation was done and V-A ECMO initiated using the *Maquet Rotaflow System*. The cannula's position was confirmed using transoesophageal echocardiogram (TOE) (Figure 2).

**Figure 3:**
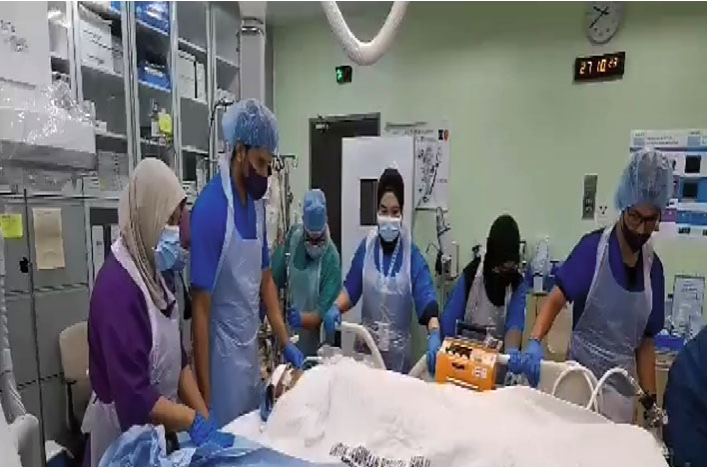
Transportation of the patient on ECMO to the cardiothoracic ICU by a critical care team led by cardiac anesthesiologists.

After the patient's hemodynamics and blood pressure were stabilized, catheter-directed thrombolysis was performed using a Judkins Right (JR) 3.5 6-Fr guiding catheter. Alteplase was administered into the left main PA, beginning with a 10-mg bolus followed by a continuous infusion of 40 mg over 18 h. Post procedure, the patient was carefully transported to the cardiothoracic ICU (Figure 3). He was kept sedated and ventilated with maximal inotropic support (noradrenaline 1 mcg/kg/min, adrenaline 1.2 mcg/kg/min, vasopressin 0.03 u/min, and milrinone 0.5 mcg/kg/min). Cerebral oximetry and advanced hemodynamic monitoring using pulse contour analysis (PCA) techniques were initiated to assist with resuscitation.

ECMO was maintained with anticoagulant heparin infusion as per protocol. Repeat bedside TOE and advanced hemodynamic monitoring revealed that the patient was suffering from right heart failure with moderate pulmonary hypertension. In view of poor hemodynamics despite full-flow ECMO, the cardiac anesthesiologist advised the cardiology team to conduct a second CDMT. Prior to the second attempt, the CTPA was re-evaluated in consultation with a radiologist. The findings suggested that the embolism in the left PA was likely chronic, which accounted for the difficulty in crossing the lesion and aspirating the thrombus. After 24 h, the cardiology team proceeded with a second attempt at CDMT using the Penumbra system, which resulted in only partial aspiration of the clot from the left PA.

Modified technique using coronary wires (0.014) with balloon inflation also attempted to open up the occluded and calcified pulmonary arteries. Unfortunately, the left upper branch was unable to be recanalized. Nebulized iloprost was started early in view of persistent pulmonary hypertension and to assist with weaning from ECMO.

During ECMO, the patient's ventilation, sedation, nutritional status, glycemic index, pressure sore care, and, most importantly, sepsis control were vigilantly managed and optimized by the anesthesiology team. His family members were constantly briefed on his progress and prognosis by the anesthesiology team. The patient was kept on ECMO for 5 days during which we slowly weaned his inotropic support with the guidance of advanced hemodynamic monitoring. ECMO cannulas were removed on Day 5, and he was successfully extubated on Day 7.

Postextubation, he was managed in the ICU for the next 3 days, during which he was counseled regarding the harm of consuming over-the-counter supplements, which could be the cause of the hypercoagulable state leading to pulmonary embolism. We also educated the patient and his family members on the importance of continued rehabilitation care, nutritional support, and compliance with his anticoagulants and other medications.

## 3. Discussion

ECMO is a form of extracorporeal life support for children and adults suffering from life-threatening cardiorespiratory failure who are resistant to conventional treatments [1]. The management of ECMO patients is inherently multidisciplinary. Cardiac anesthesiologists collaborate closely with perfusionists, intensivists, surgeons, and nursing staff to ensure comprehensive care. This teamwork is essential for successfully initiating, maintaining, and weaning ECMO support [1].

Following are several key responsibilities and interventions performed by cardiac anesthesiologists that contribute to the successful management of these patients.

### 3.1. Assessment and Selection of All Patients Referred for ECMO

Cardiac anesthesiologists evaluate the patient's overall health, comorbidities, and suitability for ECMO. Factors such as age, underlying cardiac condition, severity of illness, and potential benefits of ECMO are carefully considered and discussed with collaborating teams before initiation of ECMO.

### 3.2. Hemodynamic Monitoring and Management

Cardiac anesthesiologists play a pivotal role in the continuous monitoring of hemodynamic parameters. Invasive blood pressure monitoring is essential to maintain adequate mean arterial pressure (MAP), typically above 65 mmHg, to ensure sufficient end-organ perfusion [2]. Adjustments in ECMO flow rates, volume administration, and the use of vasopressors are common interventions to manage hypotension and maintain hemodynamic stability [2]. Pulmonary vasodilators such as iloprost are beneficial in patients with RV dysfunction with pulmonary hypertension to assist weaning from ECMO.

### 3.3. Anticoagulation Management

Effective anticoagulation is crucial to prevent thrombotic complications associated with ECMO. Cardiac anesthesiologists are responsible for the administration and monitoring of anticoagulants, primarily unfractionated heparin, to maintain target-activated partial thromboplastin time (aPTT) levels [3]. The use of thromboelastography (TEG) can provide a more comprehensive assessment of the coagulation status and guide anticoagulation therapy [3].

### 3.4. Ventilation Strategies

Managing ventilation in ECMO patients requires a delicate balance to avoid ventilator-induced lung injury. Cardiac anesthesiologists often employ lung-protective ventilation strategies, including low tidal volume ventilation, to minimize alveolar strain and overdistension [4, 5]. This approach helps in reducing the risk of further lung damage while the ECMO provides respiratory support.

### 3.5. Echocardiographic Assessment

Echocardiography is a vital tool used by cardiac anesthesiologists to assess cardiac function and guide therapeutic decisions. The use of echocardiogram can be considered for pre-, during, and post-ECMO initiation [1]. Placement and confirmation of ECMO cannulas are guided via TOE. Regular echocardiographic evaluations help in monitoring ventricular function, detecting complications such as ventricular distension, and assessing the need for interventions like left ventricular decompression [2]. Techniques such as percutaneous left ventricular assist devices or balloon atrial septostomy may be employed to alleviate ventricular overload [2].

### 3.6. Fluid and Blood Management

Optimal fluid balance management is critical in ECMO patients. Cardiac anesthesiologists carefully monitor fluid administration to avoid both hypovolemia and fluid overload [6]. Blood product transfusions are also managed judiciously to maintain adequate oxygen-carrying capacity and prevent coagulation abnormalities [2].

### 3.7. Patient Education and Rehabilitation

Anesthesiologists serve as a bridge between the medical team and the patient's family, providing updates on the patient's condition, treatment plans, and prognosis. They provide guidance on lifestyle changes and medication adherence to prevent future complications [7]. They are also the key members of the multidisciplinary team that develops and implements a comprehensive rehabilitation plan for critically ill patients following ICU admission. This process is vital to prevent impairment in a patient's physical, mental, or cognitive domains, a condition referred to as postintensive care syndrome (PICS) [8].

## 4. Conclusion

The cardiac anesthesiologist's role in managing ECMO patients is integral to achieving favorable outcomes, ensuring that patients receive the highest standard of care through meticulous hemodynamic monitoring, anticoagulation management, ventilation strategies, echocardiographic assessment, and fluid and blood management. Continuous collaboration with the multidisciplinary team further enhances the effectiveness of ECMO therapy, ultimately improving patient survival and recovery.

## Data Availability

Data sharing is not applicable to this article as no new data were created or analyzed in this study.
